# Wound Healing Potential of Chlorogenic Acid and Myricetin-3-*O*-β-Rhamnoside Isolated from *Parrotia persica*

**DOI:** 10.3390/molecules22091501

**Published:** 2017-09-08

**Authors:** Sara E. Moghadam, Samad N. Ebrahimi, Peyman Salehi, Mahdi Moridi Farimani, Matthias Hamburger, Ehsan Jabbarzadeh

**Affiliations:** 1 Department of Chemical Engineering, University of South Carolina, Columbia, SC 29208, USA; eslambol@mailbox.sc.edu; 2 Department of Phytochemistry, Medicinal Plants and Drug Research Institute, Shahid Beheshti University, GC, Evin, Tehran 1983969411 , Iran; s_ebrahimi@sbu.ac.ir (S.N.E.); p-salehi@sbu.ac.ir (P.S.); m_moridi@sbu.ac.ir (M.M.F.); 3 Division of Pharmaceutical Biology, University of Basel, Basel 4056, Switzerland; matthias.hamburger@unibas.ch; 4 Biomedical Engineering Program, University of South Carolina, Columbia, SC 29208, USA

**Keywords:** wound healing, angiogenesis, *P. persica*, myricetin-3-*O*-β-rhamnoside, chlorogenic acid

## Abstract

Wound healing is a complex physiological process that is controlled by a well-orchestrated cascade of interdependent biochemical and cellular events, which has spurred the development of therapeutics that simultaneously target these active cellular constituents. We assessed the potential of *Parrotia persica* (Hamamelidaceae) in wound repair by analyzing the regenerative effects of its two main phenolic compounds, myricetin-3-*O*-β-rhamnoside and chlorogenic acid. To accomplish this, we performed phytochemical profiling and characterized the chemical structure of pure compounds isolated from *P. persica*, followed by an analysis of the biological effects of myricetin-3-*O*-β-rhamnoside and chlorogenic acid on three cell types, including keratinocytes, fibroblasts, and endothelial cells. Myricetin-3-*O*-β-rhamnoside and chlorogenic acid exhibited complementary pro-healing properties. The percentage of keratinocyte wound closure as measured by a scratch assay was four fold faster in the presence of 10 µg/mL chlorogenic acid, as compared to the negative control. On the other hand, myricetin-3-*O*-β-rhamnoside at 10 µg/mL was more effective in promoting fibroblast migration, demonstrating a two-fold higher rate of closure compared to the negative control group. Both compounds enhanced the capillary-like tube formation of endothelial cells in an in vitro angiogenesis assay. Our results altogether delineate the potential to synergistically accelerate the fibroblastic and remodelling phases of wound repair by administering appropriate amounts of myricetin-3-*O*-β-rhamnoside and chlorogenic acid.

## 1. Introduction

Wounds are the results of injuries to the skin and can be caused by burns, microbial insults, diabetes, ischemia, and trauma [1,2]. Approximately 2% of the U.S. population is affected by chronic non-healing wounds which, along with burn victims, totals nearly 8 million people [3]. Wound repair is controlled by the cross-talk of various cytokines, growth factors, and cell types, including keratinocytes, fibroblasts, and endothelial cells [1,4]. Keratinocytes, the major epidermis cell type, initiate re-epitalization, differentiation, and migration over the injured dermis to cover the epithelial surface. Fibroblasts migrate toward the site of injury, proliferate, and establish an extracellular matrix to facilitate tissue reconstruction [5]. Endothelial cells are activated by signals expressed from keratinocytes and fibroblasts, leading to migration into the site to form new capillaries [6,7,8,9,10]. Wound contraction occurs over time in association with myofibroblasts and fibroblasts, which generate collagen and mature scars [11]. The process of wound healing can be delayed due to different agents, such as bacterial infection or an over-production of reactive oxygen species (ROS) at the site of injury, threatening live cells [12]. Using antioxidant and antibacterial agents may, therefore, improve the wound healing process and offer additional tissue protection.

Significant progress has been made in the development of therapeutics for wound closure. Commercial drugs include Regranex (Bercaplermin, recombinant platelet-derived growth factor PDGF; Johnson & Johnson), silver-based products, such as Silvadene (King Pharmaceuticals), and wound dressing gels [13,14,15]. The physiological process of wound healing is multi-factorial in nature rather than modulated by a single cytokine or cellular phenomenon. Lowering the immune response, the secretion of angiogenic growth factors, or targeting a single cellular constituent offers limited success [16]. Rather, a multi-targeted approach to modulate the interdependencies of various components of tissue regeneration is needed for clinical efficacy.

However, the development of treatments that exploit the complex network of processes involved in wound healing remains difficult and has been met with limited success so far. Natural compounds are known to interact with numerous molecular targets [17,18]. Additionally, their antioxidant, anti-microbial, and anti-inflammatory properties have attracted many laboratories to use various forms of natural compounds in wound treatment [19,20]. In this context, polyphenols and flavonoids have received significant attention due to their effectiveness in the attenuation of skin disorders and the reduction of healing time [21,22,23,24]. Researchers have variously attributed these results to (i) the inhibition of ROS [25]; (ii) the interruption of the cascade of free radical reactions [26]; (iii) the suppression of the inflammatory nuclear transcription factor kappa B (NF-κB) pathway [27]; (iv) the stimulation of collagen and elastic synthesis [28]; and (v) the reduction in membrane fluidity of bacterial cells [29].

Efforts to identify natural products with anti-inflammation and anti-bacterial properties have led to the discovery of *Parrotia persica* (*P. persica*), a member of the “Witch-hazel” family (Hamamelidaceae) [30,31]. This family contains 31 genera and more than 140 species with a broad geographic distribution [20]. This tree species was discovered for the first time by Parrot’s group [32]. Ethnobotanical studies have referred to the traditional use of this plan to treat respiratory infections and fevers. According to the literature, different extracts from the leaves of *P. persica* have shown significant antibacterial activity. Among different types of extracts, methanol extract was demonstrated to be the most potent. In addition, an analysis of the methanolic extract of *P. persica* revealed a high content of phenolic compounds, as well as a high level of antioxidant activity [32]. Other species of this family, specifically *Hamamelis virginica*, with a similar secondary metabolite profile to *P. persica*, have been demonstrated to possess anti-aging and antioxidant properties, as well as pro-stimulatory effects in keratinocytes [33,34,35]. The combination of these findings motivates our study to evaluate the wound healing potential of *P. persica*.

In the present study, for the first time, we focused on the determination of the phytochemical profile, as well as the in vitro wound healing potential of *P. persica* and its secondary metabolites. Our results demonstrate the previously unknown complementary effects of caffeoylqunic acid (i.e., chlorogenic acid) and myricetin-3-*O*-β-rhamnoside in the acceleration of wound closure and vascular tube formation using in vitro models of migration and angiogenesis. These findings reinforce the clinical evidence in support of natural compounds used alone or in combination with synthetic drugs for regenerative medicine.

## 2. Results

### 2.1. Phytochemical Profiling of P. persica

**Figure 1:**
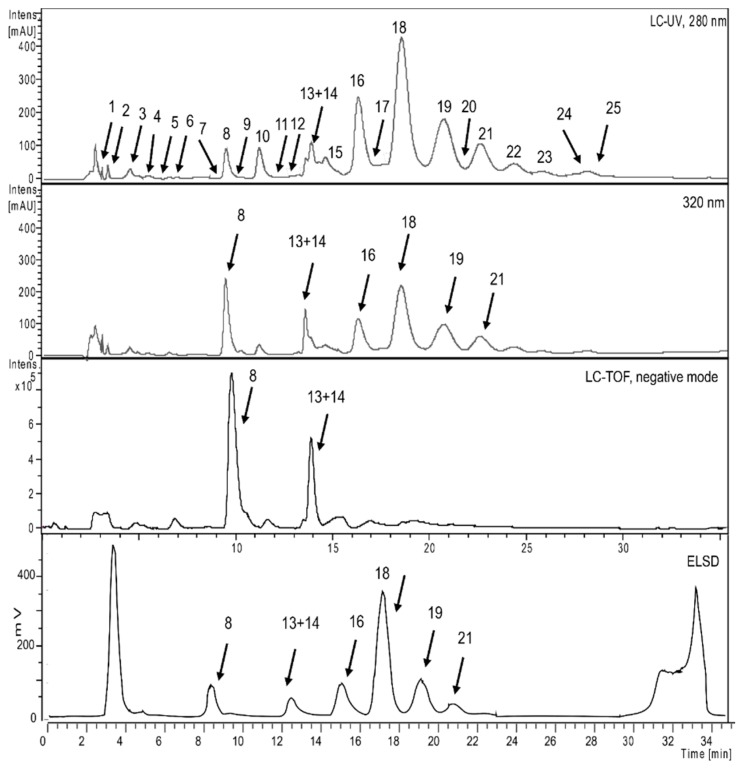
Phytochemical analysis of an EtOAc extract of *P. persica* performed using HPLC-TOF-MS combined with UV (280, 320 nm) and ELSD detection.

**Figure 2:**
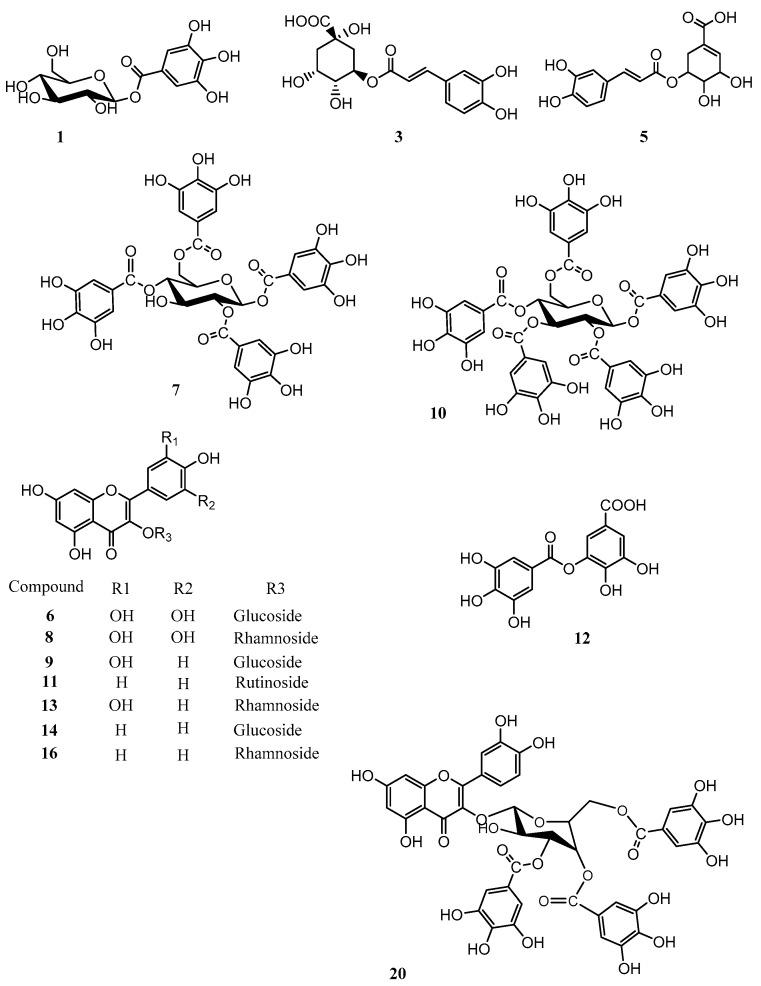
Structures of isolated compounds. Numbering is according to the chromatographic retention time.

The phytochemical profile of an ethyl acetate (EtOAc) extract of *P. persica* was analyzed by HPLC coupled with photo diode array (PDA), evaporative light scattering detector (ELSD), and electro spray ionization mass spectrometry (ESIMS) detectors. A chromatogram recorded at 280 and 320 nm, together with the ELSD trace and the ESIMS base peak chromatogram, is shown in Figure 1. It revealed a complex pattern of peaks that were both UV active and ionizable in ESIMS in the negative mode. A total of 25 compounds were detected (Table 1). Of these, 14 compounds were purified and fully characterized, while the remaining 11 compounds were tentatively identified with the aid of their molecular formula calculated from liquid chromatography time of flight mass spectrometry (LC-TOFMS) data, and by a comparison with the literature [35,36,37,38,39]. Major compounds included myricetin, kaempferol, and quercetin glycosides, as well as galloyl glucoses bearing up to fourteen galloyl residues. Caffeic acid derivatives were found as minor compounds. The UV-visible spectra (typical maxima at approx. 280 nm) and *m*/*z* values were characteristic for the presence of hydrolysable tannins [40]. The ELSD trace chromatogram of the extract corresponded with the LC-UV-ESI-TOF-MS profile, and the comparison demonstrated that tannins specifically showed that octagalloyl glucose (peak 18), and flavonoids myricetin, quercetin, and kaempferol glycosides (peak 8, 13, and 14), were the major compounds in the EtOAc extract. The chemical structures of unambiguously characterized compounds are shown in Figure 2, and tentatively identified compounds are given in Table 1 [41].

### 2.2. Selective Stimulation of Cell Growth

**Figure 3:**
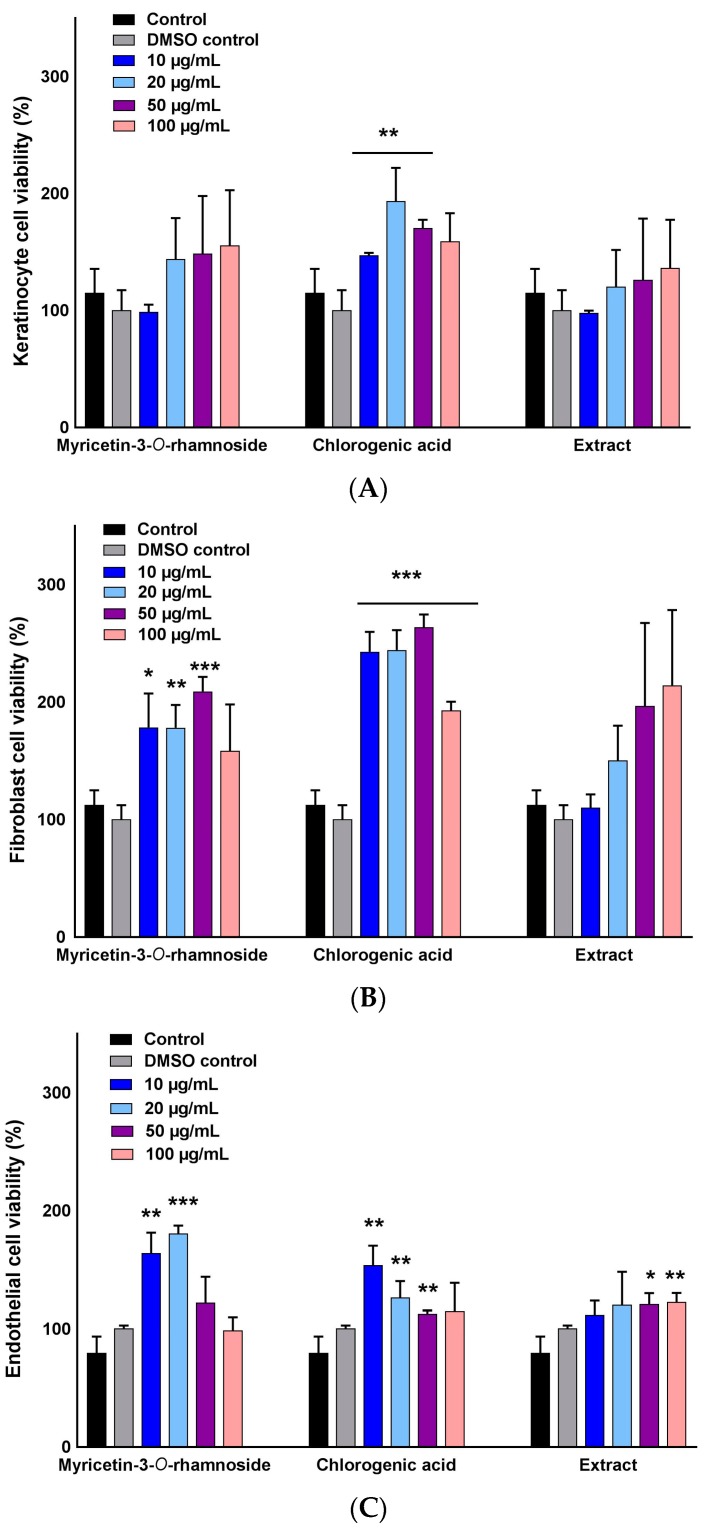
Effect of myricetin-3-*O*-β-rhamnoside (M), chlorogenic acid (C), and total extract of *P. persica* (EX) on cell proliferation of (**A**) NHEKs; (**B**) NHDFs; and (**C**) HUVECs. Cell viability (%) was calculated using an MTS assay after 24 h of exposure to various concentrations of the studied compounds. No toxicity was observed in all three cell lines when compared to the control (untreated cells in growth media) and vehicle control (untreated cells in growth media containing control DMSO). Multiple *t*-tests were performed using Graph-Pad Prism 7.03 to determine the significance between each experimental group and control (* *p* ≤ 0.05, ** *p* ≤ 0.01, and *** *p* ≤ 0.001).

We investigated the viability of a normal human keratinocyte (NHEK), normal human dermal fibroblast (NHDF), and human umbilical vein endothelial (HUVEC) in the presence of two selected pure compounds, myricetin-3-*O*-β-rhamnoside and chlorogenic acid, to determine the optimum concentrations for subsequent wound healing assays. The results were compared to control groups containing growth media, growth media plus dimethyl sulfoxide (DMSO), and growth media containing varied concentrations of *P. persica* extract (Figure 3). The viability of NHEKs was slightly concentration dependent with myricetin-3-*O*-β-rhamnoside, but no cell toxicity was seen for both compounds in the concentration range of 10–100 µg/mL (Figure 3A).

The viability of NHDFs treated with chlorogenic acid and myricetin-3-*O*-β-rhamnoside demonstrated a concentration dependent increase (10–50 µg/mL). Chlorogenic acid had a higher proliferative effect when compared to myricetin-3-*O*-β-rhamnoside and the control groups (Figure 3B). This was contrary to the pattern of HUVECs viability, indicating that the concentrations of 10 µg/mL and 20 µg/mL of myricetin-3-*O*-β-rhamnoside were the most effective dosages for cell proliferation, while 10 µg/mL was the most effective for HUVECs viability when exposed to chlorogenic acid (Figure 3C). In all cell lines, exposure to the extract at different concentrations did not result in a marked increase in cell number. Our results demonstrate the nontoxic effects of the studied chlorogenic acid and myricetin-3-*O*-β-rhamnoside on various cell lines, as well as their potential to induce proliferation.

### 2.3. Wound Healing Activity of Myricetin-3-O-β-Rhamnoside and Chlorogenic Acid

**Figure 4:**
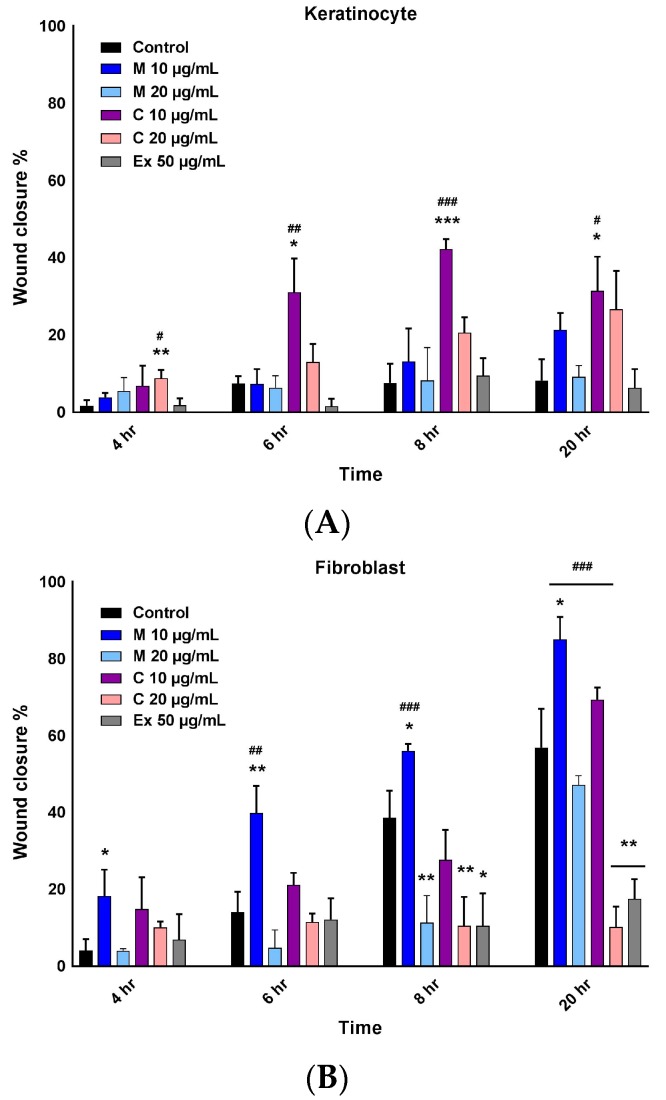
Wound closure percentage of (**A**) NHEKs and (**B**) NHDFs after different time intervals of exposure to different concentrations of the natural compounds, as measured using a scratch assay. Chlorogenic acid (C) and myricetin-3-*O*-β-rhamnoside (M) at 10 µg/mL demonstrated the highest effect on the migration of NHEKs and NHDFs in closing the gap. Multiple *t*-tests were performed using Graph-Pad Prism 7.03 to determine the significance between each experimental group and control (* *p* ≤ 0.05, ** *p* ≤ 0.01 and *** *p* ≤ 0.001). *T*-tests were performed to compare each pure compound group to extract (^#^ *p* ≤ 0.05, ^##^ *p* ≤ 0.01 and ^###^ *p* ≤ 0.001).

The ability of myricetin-3-*O*-β-rhamnoside and chlorogenic acid to accelerate the migration of NHEKs and NHDFs was evaluated using the scratch assay. Figure 4 shows the percentage of simulated wound closure over a time period of 20 h, at different time points of the assay, with two test concentrations (10 and 20 µg/mL). Controls were carried out using growth media, and growth media containing *P. persica* extract at a concentration of 50 µg/mL. Our results demonstrated that NHEKs were most sensitive to chlorogenic acid, showing the highest level of wound closure (Figure 4A). As early as 6 h, we observed approximately 40% of the gap to be closed with 10 µg/mL of chlorogenic acid. A higher concentration of chlorogenic acid, however, did not lead to a faster migration rate. Neither myricetin-3-*O*-β-rhamnoside nor *P. persica* extract led to a significantly higher NHEK gap closure than the control.

The analysis of wound closure using NHDFs demonstrated a contrasting pattern when compared to NHEKs (Figure 4B). Myricetin-3-*O*-β-rhamnoside at a concentration of 10 µg/mL accelerated gap closure at all time points. The higher concentration of myricetin-3-*O*-β-rhamnoside, however, had a negative effect on wound closure during early time points. Chlorogenic acid exhibited a delayed effect on NHDF gap closure at 20 h post-seeding. Similar to NHEKs, the extract did not accelerate the migration of NHDFs into the scratched gap when compared to the control. These results suggest a complementary effect of the two compounds in our wound healing models.

### 2.4. Pro-Angiogenic Effects of Myricetin-3-O-β-Rhamnoside and Chlorogenic Acid

**Figure 5:**
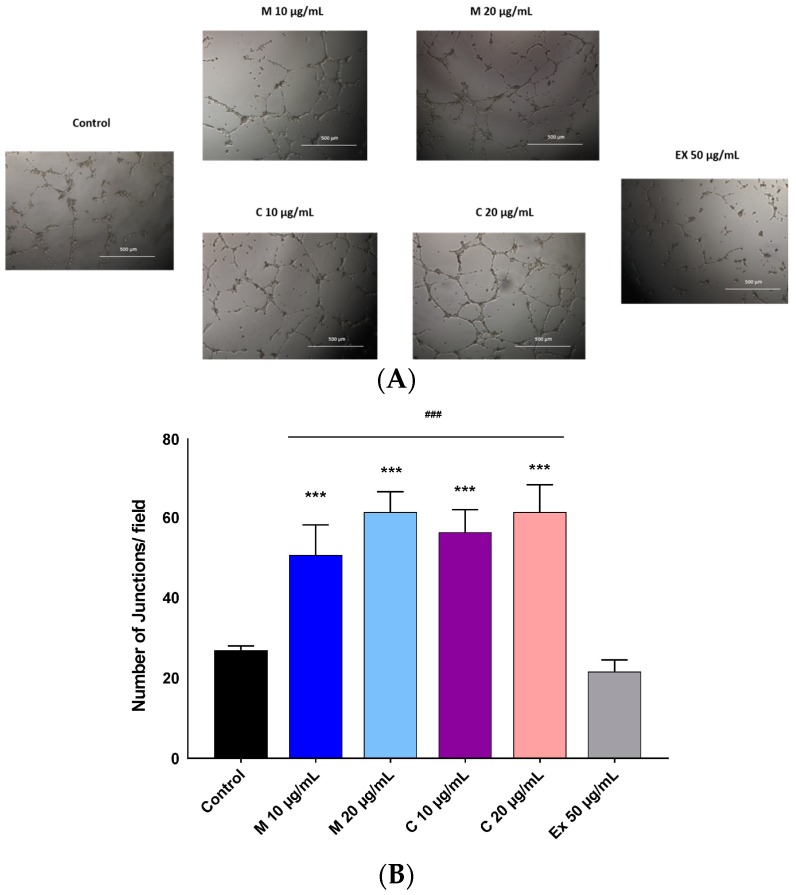
In vitro capillary tube formation of HUVECs treated in the absence or presence of myricetin-3-*O*-β-rhamnoside (M), chlorogenic acid (C), or the total extract (EX) of *P. persica.* (**A**) Phase contrast microscopy of the vascular network; (**B**) The number of junctions after 8 h of treatment. Tubular network formation was significantly higher for both the myricetin-3-*O*-β-rhamnoside (M) and chlorogenic acid compounds compared to the control (non-treated cells in growth media) and extract. Multiple *t*-tests were performed using Graph-Pad Prism 7.03 to determine the significance between each experimental group and control (*** *p* ≤ 0.001). *T*-tests were performed to compare each pure compound group to extract (^###^ *p* ≤ 0.001).

We used a capillary tube formation assay to quantify the potential stimulatory effects of chlorogenic acid and myricetin-3-*O*-β-rhamnoside on angiogenesis. The compounds were studied at two concentrations (10 and 20 µg/mL), and were added separately to HUVECs cultured on matrigels. Cells were visualized at 8 h post-seeding. Figure 5 shows the average number of junctions of the chlorogenic acid and myricetin-3-*O*-β-rhamnoside treated cultures compared to the control groups. We observed marked changes in the cell patterns, with the formation of tubules assembled by the elongation and joining of HUVECs in the presence of both myricetin-3-*O*-β-rhamnoside and chlorogenic acid (Figure 5A). A quantitative analysis of the data showed that the effect was clearly visible at 8 h when the number of junctions was approximately three-fold higher than in the control groups (Figure 5B). The results indicate a pro-angiogenic activity of the pure compounds.

## 3. Discussion

Wound healing is comprised of a cascade of biochemical events that includes three main phases of inflammation, reepithelization, and tissue remodeling [42]. The majority of innovations in drug discoveries aim at expediting the healing process. Natural compounds have garnered significant interest in drug discovery due to their multi-targeting capability, as well as their antioxidant, antibacterial, and anti-inflammatory properties [43,44]. In this context, reports have demonstrated that flavonoids (e.g., kaempferol, quercetin) and their mixtures help accelerate wound closure through a complex mechanism that involves inducing intercellular calcium-dependent pathways, stimulating collegian deposition, and the suppression of cyclooxygenase-2 (COX-2) expression [45,46]. Phenolic compounds that consist of galloyl moieties (e.g., tannins) have also been shown to be effective in wound repair and scar remodeling due to their ability to stimulate vascular formation [47,48].

In light of reports on the pharmaceutical applications of natural compounds belonging to the Hamamelidaceae family as anti-inflammatory and anti-aging drugs [30,32], we set out to elucidate the potential of *P. persica* and its main active metabolites to promote wound healing. When phytoprofiling the species, we found 25 components, including several well characterized compounds of quercetin, myricetin, and kaempherol. Interestingly, the analysis of tannin oligomers and sugar moieties in *P. persica* revealed a rich diversity in tannins featuring three to 14 galloyl units. A comparison of *P. persica’s* isolated compounds with another species of Hamamelidaceae, *H. virginica*, revealed a similar phytoconstituent profile [35]. It is important to note that *H. virginica* was traditionally used by Native Americans to treat burns and injuries [33].

The effectiveness of the Hamamelidaceae family is mainly attributed to its abundance of flavonoids and phenols, as well as polysaccharides [33,35,37,49,50]. Therefore, we chosed two under-examined polyphenol and flavonoid compounds isolated from *P. persica*, chlorogenic acid and myricetin-3-*O*-β-rhamnoside. Chlorogenic acid is an ester of caffeic acid and quinic acid, which has high bioavailability in nature [44]. Reports have shown the high potential of chlorogenic acid as an antidiabetic, antihypertensive, antitumor, and anti-inflammatory agent in the prevention of gastric lesions and hepatic injuries [51,52,53]. Caffeic acid phenetyl ester, a derivative of chlorogenic acid, has also been shown to be effective in stimulating endothelial function and vascular hypertrophy in hypertensive rats [54]. Myricetin-3-*O*-β-rhamnoside is effective in improving memory impairment in mice through inhibiting acetylcholinesterase [55]. It is also an alternative multi-target antivirulence candidate for controlling *Staphylococcus aureus* [56]. In addition, myricetin-3-*O*-β-rhamnoside prevents the photo-damage of keratinocytes caused by UV radiation [57]. These results all together motivated us to investigate the potential of chlorogenic acid and myricetin-3-*O*-β-rhamnoside isolated from *P. persica* in stimulating NHEK and NHDF migration, as well as endothelial cell capillary tube formation.

Our in vitro experiments revealed a cell-specific and dose-dependent response to chlorogenic acid and myricetin-3-*O*-β-rhamnoside. Both compounds in the range of 10–20 µg/mL were effective in promoting cell growth. We observed NHEKs to be more sensitive to the chlorogenic acid concentration, whereas the viability of endothelial cells responded more effectively to myricetin-3-*O*-β-rhamnoside. Neither compounds included a toxic effect when we maximized the dose to 100 µg/mL. Cell migration, as measured by a scratch assay, also showed variability in the cellular response to the two compounds. We observed myricetin-3-*O*-β-rhamnoside to be effective in inducing the migration of NHDFs at early time points. The mode of action was different for chlorogenic acid in that it promoted NHEK migration at early time points. Nevertheless, both compounds effectively induced capillary tube formation at a concentration of 10 µg/mL. The results suggest the role of these two polyphenol and flavonoid compounds in different stages of the wound healing process with synergistic mechanisms.

The selective impact of each compound on specific cell lines can be attributed to the different mechanisms by which flavonoids and polyphenols stimulate cells. It has been previously shown that myricetin-3-*O*-β-rhamnoside regulates the activation of mitogen-activated protein kinases and C-Jun N-terminal kinase [58]. Chlorogenic acid, however, regulates the secretion of collagens and matrix metalloproteinases [59,60,61]. It is noteworthy that the final stage of wound healing is associated with collagen secretion that regulates scar tissue formation. The most active cell types responsible in this stage of wound healing include fibroblasts and myoblasts. These cells synthesize and remodel the extracellular matrix (ECM), multiple forms of collagen, and matrix metalloproteinase (MMPs). A significant challenge in would healing strategies is to orchestrate the cascade of events that recapitulates the synthesis and remodeling of scar tissue formation. A clear advantage of natural compounds compared to synthetic drugs is that they concurrently target multiple cellular phenomena. This is particularly important in complex regenerative therapies with various cellular constituents. The complementary mode of action for myricetin 3-*O*-β-rhamnoside and chlorogenic acid is of particular interest in this study. Further studies are warranted to explore the cell-specific mechanism of action of the two compounds in co-culture, as well as in vivo conditions to define the biological contribution and mechanism of action in a more realistic wound healing model. Deeper insight into the mechanistic effects and cell signaling processes of these compounds will allow further control over cell proliferation, migration, and angiogenesis, and will improve our ability to transfer the findings of this study to clinical applications. The complex interactions of different cell types and signaling molecules in the wound healing process is ignored in this study to establish the proof of concept. Future studies should take into account these interactions in evaluating the efficacy of this strategy in vivo.

## 4. Materials and Methods

### 4.1. Chemicals and Solvents

HPLC grade water was obtained from an EASY-pure II (Barnstead, Dubuque, IA, USA) water purification system. Analytical grade solvents including petroleum ether (PetEther), ethyl acetate (EtOAc), and methanol (MeOH) for extraction and HPLC grade solvents including dimethyl sulfoxide (DMSO), acetonitrile (MeCN), formic acid (HCOOH), and methanol for chromatography were purchased from Scharlau (Barcelona, Spain). Solvents for nuclear magnetic resonance (NMR) spectroscopy were obtained from Armar Chemicals (Döttingen, Switzerland). Sephadex LH-20 was purchased from GE Healthcare (Fairfield, CT, USA).

### 4.2. Cell Culture and Reagents

HUVECs, NHEKs, NHDFs, and related media for cell cultures, including endothelial growth medium-2 (EGM-2), keratinocyte growth medium-2 (KGM™-2), and fibroblast growth medium (FGM™) were purchased from Lonza (Walkersville, MD, USA). Cells were incubated at 37 °C and 5% CO_2_ throughout the experiment. Phosphate-buffered saline (PBS), a 3-(4,5-dimethylthiazol-2-yl)-5-(3-carboxymethoxyphenyl)-2-(4-sulfophenyl)-2*H*-tetrazolium (MTS) (Promega) (Madison, WI, USA) colorimetric assay, and growth factor reduced matrigel BD (Corning), (New York, NY, USA) were purchased from Sigma-Aldrich (Milwaukee, WI, USA).

### 4.3. Instruments

NMR spectra were recorded using an Avance III™ spectrometer (Bruker BioSpin, Fällanden, Switzerland) operating at 500.11 MHz for ^1^H and 125.77 MHz for ^13^C. Spectra were obtained with a 1-mm TXI microprobe (^1^H- and 2D-NMR) and a 5-mm BBO probe with a z-gradient (^13^C). Spectra were processed with Bruker TopSpin 3.0 software (Bruker BioSpin GmbH, Rheinstetten, Germany). High resolution electrospray ionization mass spectrometry (HRESI-MS) spectra in negative ion mode were recorded on a Bruker micro time of flight mass spectrometry (TOF ESI-MS) system with a mass-to-charge ratio (*m*/*z*) scanning range of 150–1500. Mass calibration was performed with isopropanol–water (1:1) containing 5mM sodium hydroxide. The typical mass accuracy was ±7 ppm. HyStar 3.0 software (Bruker Daltonics, Bremen, Germany) was used for data acquisition and processing. HPLC-PDA-ESIMS separations were performed on an 1100 series HPLC system (Agilent, Waldbronn, Germany) consisting of a quaternary low-pressure mixing pump with a degasser module, column oven, PDA detector, and auto sampler, coupled to an Esquire 3000 plus an ion trap mass spectrometer with an electrospray interface (Bruker Daltonic, Bremen, Germany). HPLC-ELSD-PDA analysis was performed on an Alliance 2695 instrument (Waters, Milford MA, USA) equipped with a 996 PDA detector and a series 2000 evaporative light scattering detector (Alltech, Helsinki, Finland). The ELSD conditions included a nitrogen flow of 2.5 L/min and temperature of 55 °C. ESIMS spectra were recorded in the range of 150–1500 *m/z* in the negative mode.

A P50 pump (GE Healthcare, Helsinki, Finland) and fraction collector (Pharmacia Biotech, Piscataway, NJ, USA) were used for column chromatography on a Sephadex LH-20 column (870 × 70 mm). Thin layer chromatography analysis was carried out on silica gel 60 F_254_ plates (TLC) (Merck, Darmstadt, Germany), with EtOAc/MeOH/HCOOH (60/40/0.05% *v/v/v*) as the mobile phase. Detection was at UV 254 and 366 nm. For all analytical HPLC analyses, samples were dissolved in DMSO at 5 mg/mL, and 10 μL aliquots were injected. The mobile phase consisted of H_2_O and MeCN containing 0.1% formic acid. The following gradient profile with a flow rate of 0.4 mL/min was used for qualitative, LC-TOF, and ELSD analysis, starting with 20% of MeCN isocratic solution for 3 min and then an increasing gradient up to 35% MeCN over the next 3–20 min. This was followed by a 20–30 min increase to 100% MeCN and then 5 min of an isocratic solution of 100% MeCN. UV spectra were recorded in the range of 210–400 nm. SunFire^®^C_18_ (3.5 µm, 3.0 × 150 mm i.d.) (Waters, Wexford, Ireland) and SunFire C_18_ (5 µm, 10 × 150 mm) columns (Waters) were used for analytical and semi-preparative RP-HPLC separations, respectively. The column temperature was set at 30 °C.

### 4.4. Extraction and Isolation

The dried leaves of *P. persica* were powdered (90 g) and percolated sequentially with petroleum ether, EtOAc, MeOH, and H_2_O. An aliquot (7.2 g) of the EtOAc extract (17.4 g) was loaded on a Sephadex LH-20 column, which was eluted with MeOH at a flow rate of 2.0 mL/min. The column effluent was pooled according to TLC patterns to afford 16 fractions. A portion of fraction 7 (123 mg) was separated by semi-preparative HPLC using a gradient of H_2_O/MeCN (containing 0.1% formic acid) (80:20 to 100:0 over 25 min) at a flow rate of 4 mL/min to afford compounds **12** (4 mg, retention time (t_R_) 5.07 min), **6** (4.9 mg, t_R_ 8.13 min), **8** (25 mg, t_R_ 9.95 min), **13** (1.6 mg, t_R_ 12.76 min), and **11** (0.7 mg, t_R_ 17.79 min).

Fraction 5 (95 mg) was separated by semi-prep HPLC using a gradient of H_2_O/MeCN 0.1% formic acid (80:20 to 100:0 over 25 min) at a flow rate of 4 mL/min to afford compounds **8** (4.5 mg, t_R_ 9.71 min), **9** (2.7 mg, t_R_ 10.34 min), **13** (37 mg, t_R_ 11.61 min), and **17** (1.4 mg, t_R_ 15.80 min). Fraction 13 (49 mg) was separated in the same manner to yield compounds **1** (3 mg, t_R_ 3.49 min) and **3** (13.2 mg, t_R_ 6.09 min). Fraction 14 (24.8 mg) afforded gallic acid (8 mg, t_R_ 3.32 min). Fraction 15 (26.6 mg) produced compounds **5** (1 mg, t_R_ 12.1 min), **14** (9 mg, t_R_ 14.78 min), and **17** (2 mg, t_R_ 15.89 min). Fraction 16 (100 mg) afforded compounds **7** (1.6 mg, t_R_ 10.94 min), **10** (18 mg, t_R_ 11.59 min), and **20** (2.3 mg, t_R_ 24.51 min).

### 4.5. Analysis of Cell Viability

HUVECs, NHDFs, and NHEKs were cultured in their respective growth media to reach 80% confluency. Cells were seeded at a density of 5 × 10^3^ cells per well into a 96 well plate, and incubated for 24 h at 37 °C and 5% CO_2_ to allow for cell attachment. Media was changed and replaced with media supplemented with the desired concentration of test compounds (10, 20, 50, 100 µg/mL). For that purpose, stock solutions of compounds at 10 mg/mL in DMSO were diluted in culture media. The vehicle control was culture medium supplemented with 0.5% DMSO, representing the highest final concentration of DMSO used to dissolve the test compounds.

To assess cell viability following the 24 h incubation period, media containing 20% MTS solution was replaced with cell growth media, and cells were incubated for 2 h. The absorbance of formazan was measured at 490 nm using a Spectramax 190 spectrophotometer (Spectramax, Sunnyvale, CA, USA).

### 4.6. In Vitro Scratch Assay

Cell migration of different cell lines was assessed by making a scratch on confluent monolayers of NHDFs and NHEKs. Briefly, a total of 15 × 10^4^ cells per well were seeded on a 24 well plate and left overnight. Cells were given 24 h to attach and reach confluency. The cell layer was then scraped using a 200 µL pipet tip and any cell debris was washed away with PBS. The samples were then supplemented with different concentrations of compounds of interest in growth media followed by incubation in a 37 °C temperature and 5% CO_2_ controlled incubator. Images were taken every 4 h using a phase contrast Nikon Eclipse Ti-E inverted microscope. Quantification of the cell migration across the scratch to simulate wound closure was performed using the following formula:

Wound closure % = W_0_ − W_n_/W_0_

in which W_n_ is the width of the gap and W_0_ is the initial width right after creating the scratch.

### 4.7. Capillary Tube Formation

An in vitro tube formation assay was performed using HUVECs cultured on matrigel. A total of 50 µL of matrigel was added to each well of a pre-chilled 96-well plate and then incubated for 30 min at 37 °C to form a gel. Next, 100 µL of HUVEC cell suspension in media (20,000 cells/well) containing chlorogenic acid and myricetin-3-*O*-β-rhamnoside was added to the gels and incubated at 37 °C for 8 h [62]. The number of branch sites/nodes in vascular tubes was examined using a phase contrast inverted microscope (Invitrogen EVOS FL Auto Cell Imaging, Waltham, MA USA) and compared to the samples in which no compounds were added (i.e., the negative controls).

### 4.8. Statistical Analysis

Three samples (*n* = 3) were analyzed per condition unless otherwise stated. All data were statistically presented as the mean ± standard error. Multiple *t*-tests were performed using Graph-Pad Prism 7.03 (La Jolla, CA, USA) to determine the significance between each experimental group. P values less than 0.05 were considered to be significant.

## 5. Conclusions

In this study, for the first time, we analyzed the phytochemical structure of *P. persica* and its potential clinical application in the treatment of skin injuries. We found the majority of identified compounds to be tannin oligomers of three to tetradecagalloyl glucose and polyphenols. The identification of tannins with more than 10 galloyl moieties is rare among plants of this genus. The presence of these molecules confers strong antibacterial properties to *P. persica*, suggesting potential clinical use in wound regeneration applications. The in vitro analysis of the two abundant flavonoid and polyphenol molecules in *P. persica*, chlorogenic acid, and myricetin-3-*O*-β-rhamnoside, demonstrated the effective promotion of wound closure and capillary tube formation. Strikingly, the two compounds exhibited a complementary effect in that myricetin-3-*O*-β-rhamnoside accelerated fibroblast migration, whereas chlorogenic acid enhanced keratinocyte wound closure. Further studies are warranted to determine the mechanisms of action and clinical doses of these compounds using appropriate in vivo models.

## Figures and Tables

**Table 1:** List of compounds isolated and/or detected in the EtOAc extract of *P. persica*.

Peak	t_R_ (min)	Compound	UV-Vis λ_max_ (nm)	*m/z* [M−H]^−^	*m/z* [M−2H]^−2^	HPLC-TOF-MS(negative)	Identification Method
1	3.0	Galloyl glucose	231, 279	331.0657			MS-UV,NMR
2	3.5	Astringenin		405.1172			MS-UV
3	4.5	3-*O*-Caffeoylquinic acid	325	353.0861		707.1710	MS-UV,NMR
4	5.0	Trigalloyl glucose	275	635.0850		465.0706	MS-UV
5	6.2	5-*O* Caffeoylshikimic acid	288, 320	335.0781			MS-UV,NMR
6	6.5	Myricetin-3-*O*-β-glucoside	261, 357	479.0836		316.0206	MS-UV,NMR
7	8.0	1,2,4,6-Tetragalloyl glucose	275, 220	787.0996		617.0808	MS-UV,NMR
8	9.3	Myricetin-3-*O*-β-rhamnoside	260, 352	463.0887		316.022	MS-UV,NMR
9	10.4	Quercetin 3-*O*-β-glucoside	266, 356	463.0896			MS-UV,NMR
10	11.3	Pentagalloyl glucose	211, 278	939.1110		769.0952	MS-UV,NMR
11	12.0	Kaempherol-3-*O*-rutinoside		593.1525		463.0879	MS-UV,NMR
12	12.8	Digallic acid		321.0258			MS-UV
13	13.5	Quercetin-3-*O*-β-rhamnoside	258, 352	447.0935		300.0237	MS-UV,NMR
14	13.5	Kaempferol-3-*O*-β-glucoside		447.0935			MS-UV,NMR
15	14.7	Hexagalloyl glucose	214, 278	1091.1195		939.1100, 769.0865, 637.0700	MS-UV
16	16.3	Heptagalloyl glucose	214, 275	1243.1278	621.0412	1091.1181, 939,1102, 769.0859	MS-UV
17	17.1	Kaempferol-3-*O*-β-rhamnoside		431.0960			MS-UV,NMR
18	18.4	Octagalloyl glucose	214, 276	1395.1373	697.0706	1243.1265, 1091.1175, 939.1093, 767.1083	MS-UV
19	20.4	Nonagalloyl glucose	214, 276	1547.1480	773.0742	1395.1372, 1243.1268, 1091.1167, 919.1177	MS-UV
20	21.0	Quercetin-3-(3,4,6 trigalloyl glucose)		919.1236		767.1121, 615.1005, 463.0889	MS-UV,NMR
21	22.1	Decagalloyl glucose	214, 276	1699.1581	849.0767	1547.1470, 1395.134, 1243.1254, 1091.1146, 939.1083	MS-UV
22	23.7	Undecagalloyl glucose	214, 273, 305		925.0841	849.0778, 773.0715, 697.0648, 621.0590, 545.0549, 469.0500	MS-UV
23	25.2	Dodecagalloyl glucose	214, 273, 305		1001.5901	925.0841, 849.0781, 773.0721, 697.654, 621.0580, 545.0556, 469.0498	MS-UV
24	26.8	Tridecagalloyl glucose	214, 273, 305		1077.5907	1001.5907, 925.0847, 849.0779, 773.0731, 697.0649, 621.0598, 545.0554, 469.0506	MS-UV
25	27.8	Tetradecagalloyl glucose	214, 273, 305		1153.6028	1077.5960, 1001.5898, 925.0832, 849.0781, 773.0710, 697.0658, 621.0593, 545.0556, 469.0505	MS-UV
